# Meaningful patient and public engagement in dissemination—embedding co-production in dementia research

**DOI:** 10.3389/frdem.2024.1426019

**Published:** 2024-09-16

**Authors:** Susanne de Wolf-Linder, Iris Kramer, Martina Hersperger, Maria Schubert, Sonja Bächi, Monika Stolz, Emma Wolverson, Christina Ramsenthaler

**Affiliations:** ^1^School of Health Sciences, Institute of Nursing, ZHAW Zurich University of Applied Sciences, Winterthur, Zürich, Switzerland; ^2^Wolfson Palliative Care Research Centre, Hull York Medical School, University of Hull, Kingston upon Hull, United Kingdom; ^3^“Plattform Mäander” Foundation, Zürich, Switzerland; ^4^Department of Health Sciences, PPIE Stakeholder Group for People With Dementia, Institute of Nursing, Winterthur, Switzerland; ^5^Person Living With Dementia, Winterthur, Switzerland; ^6^Dementia UK, London, United Kingdom

**Keywords:** participatory research, dementia, palliative care, person-centred care, person-centred outcome measures, dissemination

## Abstract

**Background:**

Patient and Public Involvement and Engagement (PPIE) is still underutilised in both dementia research and corresponding dissemination activities.

**Aim:**

To describe the methods, format, and lessons learned in co-creating and co-producing a dissemination strategy for a research project focused on establishing patient-centred outcome measures into routine palliative community care for persons living with dementia (PLWD) and their informal carers.

**Materials and methods:**

A participatory, hybrid-format workshop was conducted to co-create the dissemination strategy with a PPIE group. A video presentation of findings and a list of prompts shared prior to the workshop were used to elicit views on dissemination strategies and knowledge translation. The workshop was followed up with a survey to consolidate the dissemination strategy. Workshop minutes and survey responses were analysed using qualitative thematic analysis.

**Results:**

22 participants from our diverse PPIE group attended the workshop. Two major themes emerged: (a) Knowledge translation: building bridges between research and practise, and (b) Collaboration and dissemination: everyone's voice is needed. Participants suggested critical changes to dissemination methods and materials. Successful knowledge translation depends on a strong evidence base. For this, materials need to be tailored to specific audiences. Everyone's voice needs to be integrated through co-production in dissemination activities by PPIE members to influence societal change. Tailored dissemination activities within a dissemination strategy were co-created spanning all phases of the research cycle.

**Discussion:**

Informing and educating the public and policymakers about the needs of PLWD relies on disseminating and fostering knowledge translation throughout all phases of the research cycle.

## 1 Introduction

Patient and public involvement and engagement (PPIE) is defined as conducting research and developing policies with or by patients and members of the public rather than on their behalf (NIHR INVOLVE, 2012). Involving members of the public in this way has been mandated by the UK Government since the late 1990s as both a core democratic principle and for pragmatic reasons (Jackson et al., 2020). Recognising the voice of those being affected by research findings and policies constitutes the moral and political principle of equity and ownership in having a say how public resources are spent (NIHR INVOLVE, 2021). It also can enhance the quality and relevance of research by including a unique perspective “from the inside” (Gove et al., 2018).

Over the past 10 years, the discourse around PPIE has changed from one of passive consultation to active involvement of people in all phases of the research cycle, ranging from conceiving relevant research questions to disseminating research findings, onto participatory research paradigms with co-production of research (Bethell et al., 2018; Burton et al., 2019; Hickey et al., 2018). As can be seen in the acronym, in its current conception PPIE focuses on three pillars: public involvement, public engagement, and participation. What must be avoided is a tokenism of involvement (Jackson et al., 2020; Hilton et al., 2024). This is partly reflected in who should be involved as members of the public. PPIE members nowadays include (potential) patients, their carers, health care professionals, but also voluntary sector workers or policy makers (NIHR INVOLVE, 2012). The aim is for researchers and the community to co-produce research that is scientifically robust, yet follows community wishes.

The incidence of dementia is increasing, affecting a substantial number of people worldwide and in European countries (World Health Organization, 2015). This has led to the European Union (EU) declaring it a priority with a view to support a rights-based approach to dementia research. However, due to its disease course of cognitive decline, people living with dementia (PLWD) have been those to whom the “right to voice” has most often been denied (Georges et al., 2022). Several national and international or European organisations and funders have tried to shift this underrepresentation by releasing position statements and standards of PPIE in dementia research (Georges et al., 2022; Gove et al., 2018). This has resulted in a growing number of research studies delivering and evaluating co-production of dementia research, potential barriers to involvement, and effective strategies to enable meaningful involvement of PPIE representatives (Bethell et al., 2018; Burton et al., 2019; Di Lorito et al., 2020; Iliffe et al., 2013; Kirby et al., 2024; Lord et al., 2022; Miah et al., 2019; Molinari-Ulate et al., 2022; Morbey et al., 2019; Poland et al., 2019; Smith et al., 2024). Meaningful involvement of PPIE representatives is of equal high value regardless the size or the focus of the study (Kirby et al., 2024; Smith et al., 2024). Involvement of PLWD and members of the public in research has been shown to support and promote a person-centred model of health care (Beresford, 2013; Collins et al., 2022; Gerlach and Kales, 2022). Three scoping reviews of PPIE involvement in dementia research conclude a tentative positive effect of such involvement (Burton et al., 2019; Miah et al., 2019; Kirby et al., 2024). However, barriers in how research is funded and organised, or barriers around researchers' and organizations' attitudes and unconscious biases have been reflected upon in qualitative and case study evaluations of PPIE in dementia research as resulting in a potentially negative effect (Bethell et al., 2018; Biddle et al., 2021; Di Lorito et al., 2020; Lord et al., 2022; Mathie et al., 2018; Mockford et al., 2016; Poland et al., 2019; Waite et al., 2019). The recruitment and long-term retention of PLWD (and not only their informal carers) in PPIE activities as well as establishing a true collaborative model of involvement and engagement are further challenges (Bartlett et al., 2019).

In dementia research, studies have developed models of co-producing research to address these challenges (e.g., the CO-research INvolvement and Engagement in Dementia (COINED) model) (Di Lorito et al., 2020; Lord et al., 2022; Mockford et al., 2016; Swarbrick et al., 2019). In these models, strategies for meaningful involvement are usually centred around the phases of a research project. These models also acknowledge the Standards of Involvement as proposed by the National Institute of Health Research (NIHR) in the United Kingdom (NIHR INVOLVE, 2012). Dissemination is defined as the active approach of spreading evidence-based findings to the target audience via determined channels using planned strategies (Tabak et al., 2012; Minogue et al., 2022). Unanimously, all studies reporting on PPIE activities in dementia research relegated these dissemination activities to the last phase of their study (Di Lorito et al., 2020; Lord et al., 2022; Mockford et al., 2016; Swarbrick et al., 2019). Some were fortunate to find some additional funds to pay for dissemination (Mockford et al., 2016) but approaches are rarely published. The only dissemination approaches identified in the literature have targeted either an academic or at least an informed audience (by PPIE members co-authoring scientific publications or co-presenting at scientific or patient organisation conferences) (Brooks et al., 2017; Utengen et al., 2017;). Direct feedback from researchers to PPIE members, particularly at the end of the study when funding might have run out (Jackson et al., 2020), is also often missing (Bagley et al., 2016; Mathie et al., 2018; Popay and Collins, 2014); and the lack of a formal evaluation of PPIE activities and their benefit to PLWD and the wider public remains an important gap in the current discourse (Mathie et al., 2018). To date, no dissemination strategy is available in dementia research that has been co-produced with PPIE and focuses on knowledge translation to the wider public.

Therefore, in this short research report we describe the methods, format, and lessons learned in co-designing and co-producing a dissemination strategy for a research project focused on establishing patient-centred outcome measures into routine palliative community care for PLWD and their informal carers. We illustrate the development of a dissemination strategy that works across all phases of the research project. Together with our diverse PPIE group involving stakeholders from different public areas, we explore novel and meaningful dissemination activities that address a wider public than is currently the case in dementia research. See Box 1 for a summary of this brief research report for the wider audience.

Box 1 **Involving people from the public, people living with dementia and people supporting a person with dementia meaningfully in research: Summary for the wider audience.**

Dementia often is not recognised enough in society. One reason for the limited recognition is that professionals often act without asking those affected by dementia. This is also true for research. Not enough people from the public, people with dementia and those supporting a person with dementia are involved or engaged in research. We wanted to address this by working together with a group of people from the community and then create a plan to share the research's findings.

Our research is about making sure people with dementia and people supporting a person with dementia get good companionship and/or care by asking them regularly about how they are feeling (e.g., are they feeling sad or are they in pain).

First, we all got together for a workshop. Some of us met in person, and some joined online. Before the workshop, we sent out a video with the findings from the research and some questions. We wanted to know how best to share these findings with a wider audience. After the workshop, we asked everyone their opinions in a survey. Then, the research team and members of the PPIE group looked at all the ideas and talked about them.

We found two big ideas: one is about making sure our research results get used in real life. The other is about making sure everyone's voice is heard when we share our findings. We learned that it is important to have good evidence when sharing our research. And we saw that it is best when everyone works together to ensure the information reaches different groups of people in easy-to-understand language.

Our plan now includes ways to share our research at every step. We believe that if we inform politicians and healthcare workers about what people with dementia need, it will make a big difference. We also believe letting people affected by dementia take the lead in disseminating this information will enhance the quality of our research. It further contributes to the inclusion/participation of people with dementia in our society.

## 2 Methods

Our research program in dementia focuses on developing, validating, and implementing person-centred outcome measures (PCOMs) into routine community care in Switzerland (de Wolf-Linder et al., 2021, 2022). Existing measures in dementia may not include outcomes important to PLWD as their perspectives are often poorly represented in the development of such measures (Morbey et al., 2019). Moreover, most measures focus on nursing home populations only, thereby inadequately reflecting the symptoms and concerns of PLWD living at home across mild to severe stages of dementia (Morbey et al., 2019; Murphy et al., 2015). Despite the inclusion of PLWD of all stages, in our research studies we conceptualise measurement of person-centred symptoms and concerns under a holistic palliative care viewpoint (Radbruch et al., 2020). Both these angles—developing a community-based and person-centred outcome measure for PLWD—have not been explored in Switzerland before. After the multi-methods development and validation of the Integrated Palliative Care Outcome Scale—Dementia for the community care setting, the research team is now co-producing a digital version of this outcome measure. The idea for this follow-on research project, the “Electronic PerSon-cENtred care and Specialised Palliative Care for people with dementIa: Improving the quality of life with Outcome guided Recognition and assessment of relevant Symptoms, neeDs and care issues” (eSENIORS) study, was voiced directly from PPIE and nurses from community/district nursing services.

Our PPIE group is embedded in the ongoing eSENIORS study (2023-2024). Participants for the group were recruited through various channels in 2023. Recruitment to this group is ongoing. We aim for a diverse range of people, including PLWD, informal carers, members of community care services, health insurance companies, public health, ethics, or health policy representatives, non-profit organisation (NPO) representatives, media experts and members of patient or dementia-related organisations e.g., Alzheimer's Society. PPIE members can represent more than one group or organisation. Most members were recruited through snowball sampling. We also promote the group, among the first of its kind in Switzerland, at public events and conferences. Individual consent for participation is negotiated via email or phone calls and re-established at the beginning of the PPIE group's activities.

### 2.1 The workshop

As part of the PPIE activities, we ran a two-hour workshop to co-design and co-produce the dissemination strategy for our research program. The workshop in December 2023 used a hybrid format of in-person attendance at our university and online attendance via a Webex board (big screen with camera). The hybrid format was agreed with the PPIE members prior to the workshop to enable inclusive opportunities according to the NIHR's standards (NIHR INVOLVE, 2012). Hybrid or online formats have been successfully employed with PPIE groups in dementia research (Brighton et al., 2018; Molinari-Ulate et al., 2022). We have followed their lessons learned to enable life conversations and interactions with all workshop participants. Three facilitators were involved in the study, the project lead (CR) and the two research associates (SdW, IK). We refrained from appointing a co-facilitator from the PPIE group due to the fact that the level of familiarity between researchers and PPIE members was not sufficiently developed at that particular moment.

All PPIE workshop participants received materials for preparation two weeks before the workshop. These included a video presentation of the study results created by the project lead and the research associate, as well as a set of questions about the presentation of results (understandability, design, style) and further avenues of knowledge translation to the public (see Table 1). We followed guidance on question prompts in communications according to the NIHR's guidance (Hickey et al., 2018).

**Table 1:** Question prompts for building the dissemination strategy.

**Prompts for considering project results:**
- Which results are particularly important to you? Why? - Who do you think needs to know about the results? - Can you think of a person who – knowing the result – would change how they act or care for PLWD?
**Prompts for considering the dissemination strategy:**
- Where should we publish the results? - Which media could we use to disseminate the results? - How could we use informal channels to distribute the findings? - Who in the group is in contact with diverse stakeholder groups? - Who would like to collaborate to make the results more accessible for everyone? - Whom, do you think, could you present the results? Who should listen to us?

In the workshop, we began with a round of introductions and clarifying expectations and setting ground rules for collaboration and co-production. Co-production of the dissemination strategy involved discussing the question prompts in small groups of four participants per table/breakout room from mixed backgrounds/groups, using first the think-pair-share method and then a world café approach (Keogh et al., 2021). Online participants were allocated in groups of four and mixed backgrounds in online breakout rooms. Both activities, think-pair-share method and the world café approach, were facilitated by the researchers. Spontaneously, one PPIE member co-facilitated the discussion at the in-person table seating the PPIE member with early-onset dementia. At the end of two rounds of discussion per table/breakout room, results were shared in the larger group and recorded on flipchart paper and—simultaneously—on a Padlet page for online attendees. The final round of discussion was followed by a casual exchange that blended formal and informal elements and concluded the workshop. We reimbursed our participants for their time per hour to prepare and attend the workshop in line with the INVOLVE guidance (Hickey et al., 2018).

After the workshop, all PPIE group members (*n* = 40), including those not able to attend the workshop (*n* = 18), were sent a survey. The survey's aim was two-fold; first, conducting a short evaluation of the first workshop and further eliciting preferences around attendance for future workshops and PPIE activities; second, confirming proposed tactics and extending ideas regarding the dissemination strategy and knowledge sharing/translation with the public. The survey link was sent out via Redcap^®^ (Harris et al., 2009). Participants could choose whether to complete the survey online or in a print-out format.

### 2.2 Analysis

A qualitative, thematic analysis and synthesis (Braun and Clarke, 2006) of both the workshop minutes and discussion notes and survey answers was undertaken by the researchers (SdW, IK). The thematic analysis focused on responses regarding the development of the dissemination strategy. We used member checking with three PPIE workshop participants (one PLWD, one managing director of an NPO, and one nurse) to validate and extend results.

## 3 Results

Twenty two participants attended the workshop, 15 in person and 7 online. See Table 2 for the profile of participating PPIE group members. Comments in the survey were received from 24 participants. Five survey participants were unable to attend the previous workshop and therefore responded only to strategical questions with regards to the dissemination strategy. Overall, the workshop was evaluated as a positive activity for those attending. Several adjustments for making PPIE contribution an inclusive opportunity were described by survey respondents.

**Table 2:** Profile of PPIE group members (*n* = 40; 4 double roles^*^), attendees at the workshop (n=22; 1 double role^**^), and participants providing answers to the survey (*n* = 24; 3 double roles^***^).

**Roles (*n*)**	PPIE group (*n* = 40^*^)	Workshop (*n* = 22^**^)	Survey (*n* = 24^***^)
Person living with dementia	2	1	1
Family member	10	6	7
**Nurses**			
Community care	10	6	5
Acute care (geriatrics)	5	2	3
Geriatric/dementia counselling	3	2	3
District nurse union	1	1	1
Support group manager	2	-	-
Social counselling	1	-	-
**NPOs for dementia, geriatric associations**			
Managing director NPO	1	1	1
Senior citizens organisation	1	-	1
Church community	1	1	1
Public relations (journalist)	1	1	-
Alzheimers Association	1	-	1
Cultural club	1	1	1
Gerontological association	1	1	1
Politician	1	-	-
Community administration	1	1	1

Two major themes emerged regarding how best to achieve a collaborative model of involvement and engagement in disseminating research: (a) Knowledge translation: Building bridges between research and practise, (b) Collaboration and dissemination: Everyone's voice is needed. We lastly present a dissemination strategy that integrates into all phases of the research cycle.

### 3.1 Knowledge translation: building bridges between research and practise

PPIE participants needed encouragement to voice critical views on the materials received. Participants suggested small changes to the prepared dissemination materials which can be summarised under the heading “less is more”. For instance, they felt we needed to tailor information materials to the intended audience by focusing on one message per slide in presentations and choosing a simpler colour scheme. For a successful knowledge translation reaching a diverse range of audiences, participants suggested a different use of language and alerted to the use of technical terms and jargon that might be differently understood by different audiences. However, participants were adamant about the need to be evidence-based in their dissemination:

“*Research is part of everyday life”* (Advanced nurse practitioner, Geriatric/dementia counselling)

To achieve knowledge translation into everyday life, they suggested support from non-academic writers to avoid jargon in dissemination materials like newspaper articles or flyers. Once trust was built among members of the workshop, participants felt comfortable to take control of the dissemination. They suggested developing larger communication programs (e.g., a series in newsletter format) to disseminate implications for practise and research.

### 3.2 Collaboration and dissemination: everyone's voice is needed

Participants voiced concerns about the power imbalance when researchers communicated to non-academic audiences. Several ideas around co-presenting or sole facilitation/dissemination by a lay member were brought forward to reach diverse audiences. Several of our group members (particularly informal carers) stepped up during the worldcafé to spontaneously co-facilitate the discussion at their table. Some PPIE members also helped each other while preparing for the workshop. With the facilitation of a community nurse familiar to her, our PLWD member was able to contribute important insights for both designing dissemination materials tailored to PLWD and the importance of a joint dissemination/communication strategy uniting all voices. The group felt that given dementia is often perceived as a Cinderella disease, isolating, and rendering those affected by it almost invisible, everyone is needed to contribute to research findings to be heard:

“*From backyard thinking to network thinking—that's the mission!”* (PR for dementia and geriatric association)

### 3.3 Dissemination strategy to communicate results in dementia research

**Figure 1:**
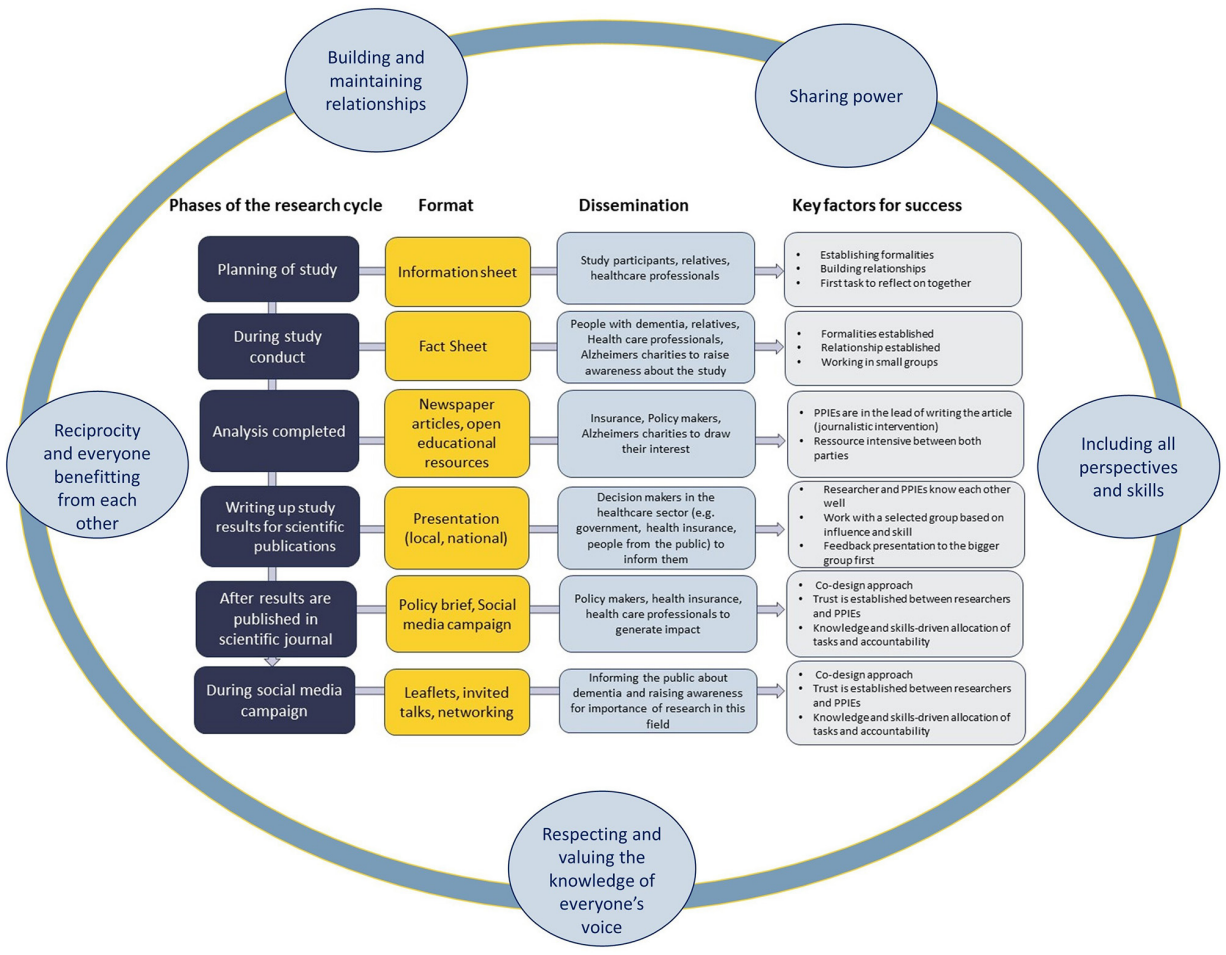
Dissemination strategy integrated into all phases of the research cycle embedded in the key principles of the NIHR guidance on co-producing research (NIHR, 2019).

At the end of the workshop and with the help of consolidation via the online survey, we agreed on a dissemination strategy traversing the whole research cycle. In order to reach different audiences and for everyone to be able to contribute, participants suggested to integrate dissemination strategies and knowledge translation throughout all phases of a research project. Figure 1 summarises a range of strategies to reach academic and non-academic audiences and the general public, the target of the dissemination activities, and key factors for success.

## 4 Discussion

Using a co-production workshop with members of our PPIE group, we have developed a dissemination strategy that transcends all phases of the research cycle. Unlike common models of integrating PPIE activities into a study, we propose for dissemination to become an integral part of the research lifecycle, not just at the end of the study when it might be difficult due to time and funding constraints to reach meaningful involvement and engagement of PPIE (Bate and Robert, 2006; Greenhalgh et al., 2019; Kirby et al., 2024). Based on our findings, we propose for dissemination and knowledge translation to be considered activities of co-production rather than mere person or user-centred traditional approaches of consultation (Jackson et al., 2020). Collaborating as equal partners while recognising and valuing diverse knowledge, experiences, social networks, and cultural methods, are essential moral principles that should guide individuals engaged in co-productive activities (Jackson et al., 2020). Ideally, these dissemination activities are organised according to the key principles of the NIHR guidance on co-producing research (NIHR, 2019)—(a) sharing power, (b) including all perspectives and skills, (c) respecting and valuing the knowledge of all those working together with equal importance of everyone's voice, (d) reciprocity and everyone benefitting from each other, and (e) building and maintaining relationships as a means to share power. Embedding such a dissemination strategy (Figure 1) into the overall PPIE strategy can directly benefit the research project, e.g., representing the project as a lay member at the ethics committee review meeting, reviewing and adapting patient information leaflets or writing a lay summary. Such dissemination strategies can draw on and benefit from the unique inside perspective of PPIE participants, and their diverse skill set, experiences, and social networks. For this to be successful, the NIHR's (2019) principles need to be followed. This can then build the collective confidence of the PPIE group. PPIE members in our workshop group were cognizant of both the power of their voice and the right to express that voice as a political means to confirm the personhood of PLWD in society.

Through the feedback in our workshop, we have also realised that a view of framing PPIE as co-production in both research and dissemination may be too high a demand in a PPIE-naïve country without funding infrastructure such as Switzerland (Biddle et al., 2021; Miah et al., 2019), an aspiration and goal rather than a reality. Similar to what is concluded in existing scoping reviews of PPIE co-production in dementia research, there also remains a need for the thorough evaluation of PPIE activities, also capturing less positive or overwhelming experiences with PPIE reported from all perspectives (Hendriks et al., 2017; Russell et al., 2020). The members of our PPIE group were eager to transform less positive experiences from the workshop (e.g., feeling overwhelmed by too much material, researchers talking to long about research findings, reacting spontaneously to new material, public speaking) into valuable learning opportunities for future workshops by assuming responsibility for driving positive change within the group. While our PPIE group members also remained very keen on contributing to the study and the dissemination of its findings, barriers to meaningful, sustainable contribution were also voiced. Many of the issues around time constraints, conflicting care obligations, money and reimbursement issues, and worries about committing long-term to the group may also reflect the socioeconomic disadvantage of belonging to a group often marginalised in Western societies (Biddle et al., 2021; Miah et al., 2019). As part of the workshop and its evaluation, participants have also suggested ways to address these barriers (see Table 3). We have categorised the suggestions around the six NIHR standards of involvement (NIHR INVOLVE, 2019). In addition, we have embedded suggestions from the literature on how to achieve meaningful engagement and co-production via PPIE in dementia research (Bagley et al., 2016; Burton et al., 2019; Ferra et al., 2023; Georges et al., 2022; Gove et al., 2018; Hilton et al., 2024; Jackson et al., 2020; Kirby et al., 2024; Lord et al., 2022; Masoud et al., 2021; Mathie et al., 2018; Miah et al., 2019; Morbey et al., 2019; Poland et al., 2019; Popay and Collins, 2014; Smith et al., 2024; Staniszewska et al., 2017). Many of these suggestions are novel in the sense that they focus on how to engage PPI members in dissemination activities, rather than focusing on how to engage them in dementia research. However, these more general recommendations also apply to engaging them in dissemination activities.

**Table 3:** Addressing the NIHR's six standards of involvement around PPIE in dissemination with lessons learned.

**NIHR's standards of involvement (NIHR INVOLVE, 2019)**	**Explanation**	**Identified risk factors for achieving meaningful PPIE involvement in dissemination**	**Lessons learned for meaningful engagement in dissemination activities**
Inclusive opportunities	Offer public involvement opportunities that are accessible and that reach people and groups according to their needs	- Risk of information overload, feeling overwhelmed by too much information - Cost barriers - Tokenism and using PPIE involvement as an afterthought - Venue selection and accessibility - Meeting schedules and manners of involvement not meeting the needs of various stakeholder groups - Communication challenges - Overprotection and limitation of engagement - Time constraints	- Integrating PPIE-led and co-produced dissemination activities throughout the research project and not confining it to the last phase of the project. - Co-developing meaningful activities around sharing research findings as well as engaging the wider community. - Involving everybody in a manner that they find meaningful. - Members of the PPIE group working in pairs, peer support as key. - Sending study-related questions and information ahead of the workshop and involving members of the PPIE group - Flexibility around meeting times and manner of involvement, following a person-centred approach - Planning additional costs, also around co-facilitation and running meetings in an inclusive way - Pragmatism and compromise - Ongoing engagement and recruitment - Open format engagement - Accessibility and dementia-friendly formats, short communication
Working together	Work together to value all contributors, and that builds and sustains mutually respectful and productive relationships	- Lack of person-centred approach - Limited choices and adaptability - Insufficient group building - Neglecting multiple viewpoints - Inadequate support and training - Power imbalance and role ambiguity	- Researchers and members of the PPIE group openly discuss the duration of their commitment. Various forms of commitment, such as those that incorporate breaks, may emerge and require consideration and integration - Prioritising well-being and choice - Build rapport and equality, establish a buddy system and peer support in the group - Include diverse viewpoints and diverse smaller groups to engage with certain dissemination activities - Provide support and training to all members, use co-facilitation in training sessions - Encourage mutual understanding and learning - Establish clear roles and responsibilities, but keep them short term and tailored to the individual dissemination activity - Promote cooperative management structures - Engage the community and with the wider societal views of dementia to combat the cinderella status of dementia
Support and learning	Offer and promote support and learning opportunities that build confidence and skills for public involvement	- Lack of informal environment - Neglecting carer support and guidance - Lack of communication training for researchers - Uncertainty and anxiety around contributing to research - Emotional toll on researchers and PPIE	- Substantial time should be allocated to identify support and learning needs from everyone in the PPIE group - Using informal meeting components to address anxieties, create an informal environment - Researchers and PPIE members to co-plan meetings and learn about needs, facilitate pre-meetings - Offer specific communication training for different groups and use PPIE members to co-facilitate training - Making sure to plan meetings and engagement with carer support in mind, also supporting carers to support the PLWD - Engage PPIE members to create resources (e.g., short videos) what PPIE work is about - Prioritise knowledge assimilation and cultural understanding
Governance	Involve the public in research management, regulation, leadership and decision making	- Lack of clarification and documentation of how PPIE input is used in the dissemination strategy - Insufficient monitoring of activities - Lack of formal governance structure leading to inconsistencies in decision-making, and potential biases	- Researchers ought to allocate time and resources to draft clear and concise codes of conduct using accessible language, involving PPIE members - Document involvement processes - Lobby and co-design the governance structure, build in monitoring activities and frequent feedback to make sure that all processes align with the standards
Communications	Use plain language for well-timed and relevant communications, as part of involvement plans and activities	- Excluding relevant stakeholders - Misunderstandings and tensions in PPIE activity, unmet expectations and failure to recognise that - Inconsistencies in seeking input from PPIE contributors - Lack of training around appropriate communication for researchers	- Researchers transfer the lead for communications to members of the PPIE group to ensure that the message is conveyed in a manner that is easily comprehensible to the intended audience - Provide consistent and supportive guidance for PPIE contribution - Check on mutual understanding of tasks and involvement/engagement, recognise PPIE activities as a site of multiple understandings - Invest in training - Understanding the audience and produce targeted resources for the intended audience
Impact	Seek improvement by identifying and sharing the difference that public involvement makes to research	- Lack of formal evaluation making it difficult to assess the benefits and the effectiveness - Lack of frequent feedback loops, lack of focusing on learning from negative experiences, lack of assessing potential negative experiences among PPIE members - Inadequate resources for monitoring and evaluation - Limited reporting of PPIE impact in dissemination - Absence of standards for evaluating PPIE quality	- Formally evaluate the effectiveness and impact of PPIE involvement in dissemination - Frequent evaluations and feedback loops engaging all members in a format and to an extent that is appropriate and meeting needs - Plan and allocate sufficient resources for evaluation - Systematically reporting PPIE impact in all activities - Co-creating standards of involvement and how to best evaluate them

We acknowledge that in the workshop, we only had one PLWD attending. In our PPIE group, we currently have two PLWD participants. It has been acknowledged that recruitment and retention of PLWD to PPIE activities remains a challenge (Masoud et al., 2021; Moreno et al., 2023). Our workshop did not include co-facilitation by PPIE members as our primary focus was on exploring the expectations and visions of the group regarding their involvement and establishing a basis for our work. As dementia-aware facilitators, we appreciated that the group was diverse in their needs (Masoud et al., 2021). By using group work techniques that facilitated peer support and hearing diverse voices we hoped to develop a co-created code of conduct with shared values, beliefs, and attitudes. However, with the group now being initiated, and with the ongoing recruitment of new members, we are planning to explore avenues for co-facilitation to better consider the needs of PPIE group members, particularly around avoiding information-heavy meetings. Lastly, as academic researchers we also acknowledge the need for further training around effective communication and facilitation strategies of workshops with a diverse range of people from different backgrounds attending.

Limitations to this work apply. Although we analysed our study using principles of qualitative thematic analysis, the manner of sampling, data collection, and analysis cannot be considered representative of a qualitative study. We share anecdotal evidence of what worked in our project. The representativeness of our findings is limited.

## 5 Conclusion

We have developed a dissemination strategy with a diverse PPIE group, including PLWD and informal carers. In every dissemination activity, we advise to tailor the illustration, language format, and overall message to a specific target audience and working with PPIE group members to co-produce disseminiation materials. By sharing or even handing over the lead in dissemination activities, we believe that knowledge translation can be fostered and that research findings can reach those audiences that can bring about a change in public health and societal views around the stigma associated with dementia (Low and Purwaningrum, 2020). Our results provide new avenues of how and when to disseminate research findings to maximise their impact.

## Data Availability

The raw data supporting the conclusions of this article will be made available by the corresponding author upon reasonable request.
